# Prevalence and predictors of hypotension on hospital arrival in traumatic brain injury: a prehospital HEMS cohort study

**DOI:** 10.1038/s41598-026-45208-7

**Published:** 2026-03-22

**Authors:** Agnė Macaitė, L. S. Scholl, J. Schwietring, S. Rehberg, A. Hoyer, K.-C. Thies

**Affiliations:** 1 Medical School and University Medical Centre OWL, Department of Anaesthesiology, Intensive Care, Emergency Medicine, Transfusion Medicine and Pain Therapy, Bielefeld University, Protestant Hospital of the Bethel Foundation, Bielefeld, Germany; 2 Gerhard-Domagk-Institute of Pathology, University Hospital Münster, Münster, Germany; 3 ADAC Luftrettung gGmbH, ADAC Luftrettung gGmbH, München, Germany; 4 Medical School OWL, Biostatistics and Medical Biometry, Bielefeld University, Bielefeld, Germany

**Keywords:** Traumatic brain injury, Hypotension, Prehospital care, Emergency medicine, Diseases, Medical research, Neurology, Neuroscience, Risk factors

## Abstract

Hypotension is a well-established predictor of poor outcomes and increased mortality in patients with traumatic brain injury (TBI). Even a single episode of low systolic blood pressure (SBP) during the prehospital phase is associated with a worse prognosis. To minimise secondary brain injury, early identification and management of hypotension are critical. While international guidelines increasingly recommend higher SBP thresholds in TBI care, structured prehospital data remain limited in the German emergency care context. This study aimed to quantify the prevalence of hypotension on hospital arrival among adult TBI patients transported by helicopter emergency medical services (HEMS) and to identify high-risk subgroups. This retrospective cohort study analysed ADAC Luftrettung mission data from 2017 to 2021. Adults (≥ 18 years) with documented TBI in the mission record were included. Hypotension was defined as SBP < 90 mmHg in line with current German guidance. Two time points were assessed: SBP at initial HEMS contact (“initial hypotension”) and SBP on hospital arrival (“hypotension on hospital arrival”). TBI severity was classified by Glasgow Coma Scale (GCS), and injury patterns were recorded as isolated TBI, multiple injuries (non-polytrauma; “Mehrfachverletzung”), or polytrauma. A multivariable logistic regression was used to identify independent predictors of hypotension on hospital arrival. A total of 20,756 patients were included (67.7% male; median age 55.0 years). Hypotension on hospital arrival occurred in 3.4% of patients overall and in 35.5% of those with initial hypotension. Initial hypotension was the strongest predictor of hypotension on hospital arrival (OR 13.82, 95% CI 11.47–16.65). Severe TBI (OR 4.26, 95% CI 3.41–5.32) and polytrauma (OR 3.08, 95% CI 2.44–3.90) were additional independent predictors. Initial hypotension identifies a high-risk subgroup of adult TBI patients transported by HEMS who are substantially more likely to be hypotensive at hospital arrival, particularly those with severe TBI and polytrauma. These findings support prioritising early haemodynamic stabilisation in this population and provide a basis for future outcome-linked studies in the German setting.

## Manuscript

 In Germany, an estimated 300–400 per 100,000 individuals sustain a traumatic brain injury (TBI) annually^1^. Despite the clinical and socioeconomic burden, comprehensive prehospital data remain limited^2^.

Hypotension is a well-established predictor of poor outcomes in TBI, including increased mortality, neurological impairment, long-term disability, and extended intensive care stays. A 2024 meta-analysis of nearly 400,000 patients found a significantly increased risk of death among TBI patients with hypotension^3^. Further evidence indicates that hypotension occurring in the field or persisting until hospital arrival is associated with the highest mortality risk^4^. While primary brain injury is irreversible, secondary injury may be mitigated by maintaining adequate cerebral perfusion through appropriate blood pressure management^5^.

Current German guidelines define hypotension in TBI as systolic blood pressure (SBP) < 90 mmHg^6,7^. International recommendations increasingly advocate higher prehospital SBP targets, as observational data indicate a largely monotonic relationship between lower SBP and mortality across a broad range rather than a single threshold. In the EPIC cohort, prehospital SBP was associated with in-hospital mortality across the range studied, suggesting that clinically relevant hypoperfusion may occur above 90 mmHg and supporting recent recommendations to target SBP ≥ 110 mmHg^8,9^.

Given the evolving evidence and the absence of structured national prehospital data in Germany, this study aimed to assess the prevalence of hypotension among adult TBI patients transported by physician-staffed HEMS and to identify independent prehospital predictors, with particular focus on injury severity and pattern. As hypotension duration cannot be determined, we used SBP on hospital arrival as the primary endpoint, reflecting haemodynamic status at handover; admission SBP is associated with mortality in significant TBI^10^. In this setting, SBP < 90 mmHg on arrival indicates unresolved haemodynamic instability despite prehospital management and identifies a high-risk subgroup for quality improvement and future outcome-linked research.

## Methods

### Study design

This retrospective cohort study analysed routinely collected operational data from ADAC-Luftrettung, a physician-staffed helicopter emergency medical service.

### Setting

HEMS dispatch in Germany is regionally organised and typically prioritises suspected life-threatening injury or critical illness (e.g., severe mechanism, altered consciousness, physiological derangement, complex extrication, remote location, or anticipated need for physician-level interventions). Dispatch decision variables were not available in the mission dataset; therefore, the cohort is described as adult TBI patients transported by HEMS rather than as a population-based TBI sample.

### Data source

Data were extracted from the ADAC-Luftrettung electronic mission log, which is completed prospectively by the HEMS team immediately after each mission and contains prehospital clinical and operational variables only, without in-hospital diagnostics, treatments, or outcomes. The logs cover the period from 2017 to 2021; ADAC Luftrettung is one of Europe’s largest HEMS providers and completed over 200,000 missions during the study period.

### Population

**Fig. 1:**
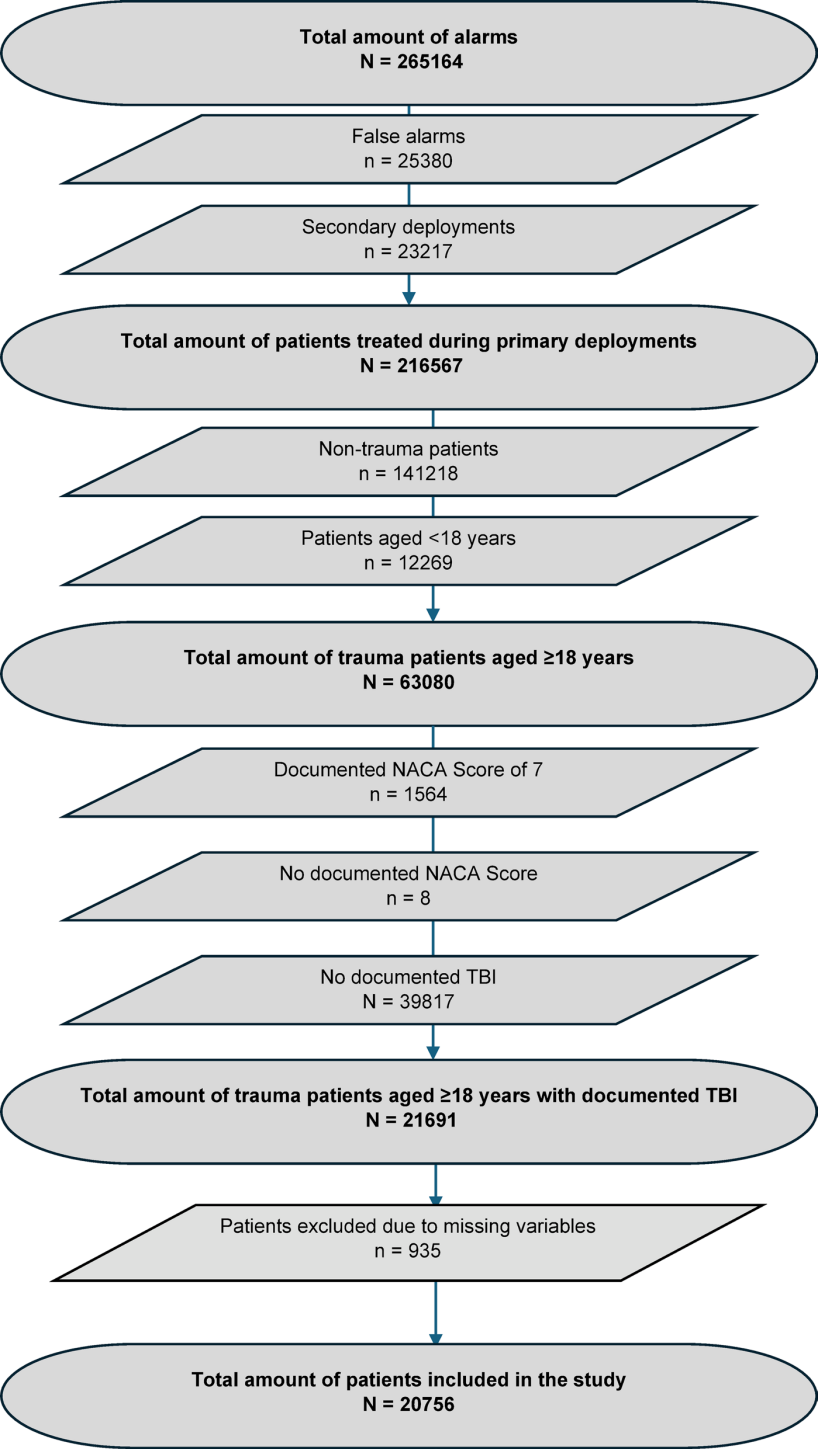
Study population selection process.

All adult patients (≥ 18 years) with documented traumatic brain injury (TBI) in the electronic mission record were eligible. Patients with missing data for key variables (GCS category, injury pattern, or systolic blood pressure at either time point) were excluded (complete-case analysis). The patient selection process and exclusions are summarised in Fig. 1.

### Outcomes

Hypotension was defined as systolic blood pressure (SBP) < 90 mmHg, in accordance with current German guidelines^6^, which apply this threshold as a general criterion for hypotension in TBI rather than stratifying it by Glasgow Coma Scale (GCS) category. We therefore used SBP < 90 mmHg as the operational definition across all TBI severities in our analysis, while acknowledging ongoing debate and higher thresholds proposed in recent international recommendations. SBP at initial HEMS contact was defined as initial hypotension, and SBP recorded at hospital handover as hypotension on hospital arrival (primary endpoint). As SBP was available at two discrete time points only, hypotension on hospital arrival reflects haemodynamic status at handover and does not quantify duration of hypotension. TBI severity was categorised using the Glasgow Coma Scale (GCS) as mild (GCS 13–15), moderate (GCS 9–12), or severe (GCS 3–8)^11^. Injury pattern was recorded prospectively in the mission documentation and classified as: (i) isolated injury, (ii) multiple injuries (non-polytrauma; Mehrfachverletzung), defined as more than one injury without an overall life-threatening injury constellation as assessed by the HEMS physician, and (iii) polytrauma, defined as an injury constellation recorded as life-threatening in the mission report. These categories were analysed as documented and were not derived post hoc from AIS/ISS, which are not available in the mission dataset. To avoid semantic ambiguity in English, we use the term “multiple injuries (non-polytrauma; Mehrfachverletzung)” for the intermediate category. This categorisation aligns with the German Trauma Registry and with standard prehospital documentation in Germany^6,12^.

### Statistical analysis

Data were analysed descriptively according to variable type, using medians with interquartile ranges for continuous variables and counts with proportions for categorical variables. To identify variables independently associated with hypotension on hospital arrival, we fitted a single multivariable logistic regression model including initial hypotension (yes/no), age, sex, injury pattern, and TBI severity (GCS categories). Covariates were selected a priori based on clinical relevance and availability in the dataset. Effect estimates are reported as odds ratios (ORs) with 95% confidence intervals (CIs) and p-values as appropriate. A p-value ≤ 0.05 was considered statistically significant. All analyses were performed using SAS 9.4 (SAS Institute Inc., Cary, NC). No prospective protocol was registered, and no a priori sample size calculation was performed because all eligible cases within the study period were included.

## Results

### Cohort characteristics

Fig. 1 summarises cohort selection. During the study period there were 265,164 alarms, including 25,380 false alarms and 23,217 secondary deployments. In primary deployments, 216,567 patients were treated; 141,218 were non-trauma, 12,269 were < 18 years, and 1,572 had a documented NACA score of 0 or 7. Of 63,080 adult trauma patients, 935 were excluded due to missing key variables and 39,817 had no documented TBI, leaving 20,756 adult trauma patients with documented TBI for analysis. 14,043 (67.7%) were male and 6,713 (32.3%) were female. The median age was 55,0 years (interquartile range [IQR]: 37.75–72.08). Injury patterns were classified as follows: 5,849 patients (28.2%) sustained isolated TBI, 9,479 (45.7%) had TBI in the context of multiple injuries, and 5,428 (26.1%) sustained TBI as part of polytrauma. Regarding TBI severity, 14,909 cases (71.8%) were mild, 1,853 (8.9%) moderate, and 3,994 (19.2%) severe.

An overview of the cohort characteristics is provided in Tables 1 and 2.

### Hypotension prevalence

Hypotension on hospital arrival was observed in 3.4% of the cohort. Among patients presenting with initial hypotension, 35.5% developed hypotension on hospital arrival, compared to only 1.4% in those with normal initial SBP.

Hypotension on hospital arrival was more frequent in severe TBI, particularly when combined with polytrauma. In this subgroup 7.0% of patients with isolated TBI, 5,0% with multiple injuries, and 19.6% with polytrauma developed hypotension on hospital arrival.

Among patients with moderate TBI, hypotension on hospital arrival was observed in 1.6% of isolated TBI,1.3% with multiple injuries, and 7.0% with polytrauma.

In mild TBI, rates were lower: 0.5% in isolated injuries, 0.4% in multiple injuries, and 3,0% in polytrauma cases.

Full subgroup data are presented in Table 2.

### Logistic regression analysis

Across all TBI severities, polytrauma was consistently associated with a higher likelihood of hypotension on hospital arrival. Logistic regression confirmed **initial hypotension** as the strongest predictor (odds ratio [OR] 13.82; 95% confidence interval [CI]: 11.47–16.65). Additional independent predictors included **Severe TBI** (OR 4.26; 95% CI: 3.41–5.32), and **Polytrauma** (OR 3.08; 95% CI: 2.44–3.90).

**Fig. 2:**
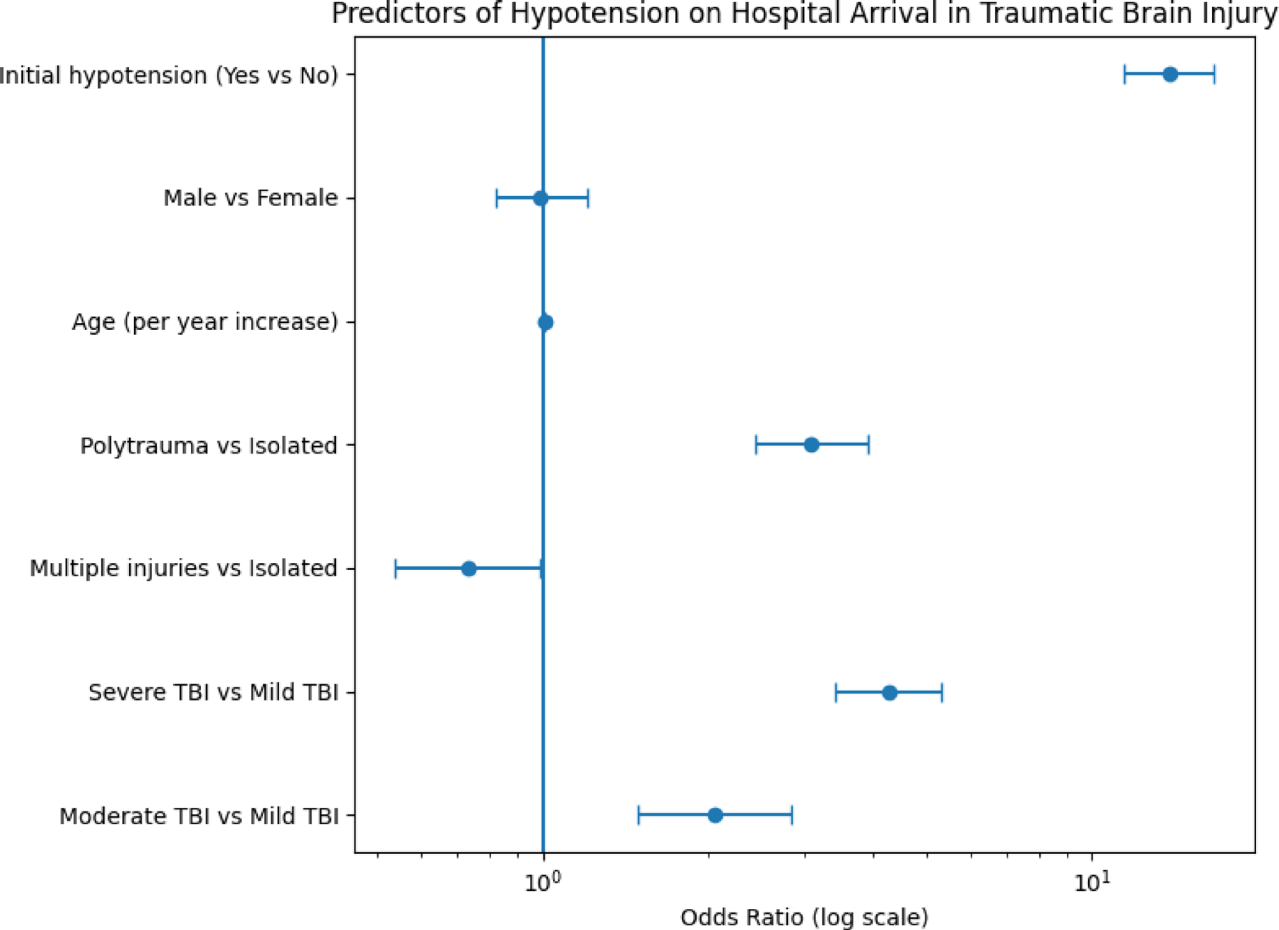
Forest plot of multivariable logistic regression predicting hypotension on hospital arrival in patients with traumatic brain injury.

The full results of logistic regression model predicting hypotension on hospital arrival in patients with TBI, depending on variables are presented in the Table 3; Fig. 2.

As an exploratory analysis addressing emerging international targets, SBP < 110 mmHg on hospital arrival occurred in 11.3% (2,489/22,028) of TBI patients overall and was most frequent in severe TBI with polytrauma (37.2%; 803/2,160), with consistently lower proportions in isolated TBI and in less severe TBI categories; in a corresponding multivariable model, the pattern of independent predictors mirrored the primary SBP < 90 mmHg analysis (Supplementary Tables 1 and Supplementary Table 2).

**Table 1:** Cohort characteristics.

Variable	Patients with initial hypotension (SBP < 90mmHg) (*n* = 1239)		Patients without initial hypotension (SBP ≥ 90mmHg) (*n* = 19517)		Total (*n* = 20756)
**Age**					
Mean (SD)	57.6 (20.30)		54.9 (20.83)	55.0 (20.81)	
**Sex**					
Male	888 (71.7)	13,155 (67.4)		14,043 (67.7)	
Female	351 (28.3)	6362 (32.6)		6713 (32.3)	

**Table 2:** Prevalence of hypotension by TBI and injury pattern. Multiple injuries’ refers to the mission-documented category ‘Mehrfachverletzung’ (non-polytrauma) and is presented separately from ‘polytrauma’ as recorded in the dataset.

Severe TBI	Isolated TBI	Multiple Injuries	Polytrauma
	1236 (30.95%)	803 (20.10%)	1955 (48.95%)
BP<90mmHg (initial)	175 (14.16%)	108 (13.45%)	601 (30.74%)
BP<90mmHg (hospital arrival)	87 (7.04%)	40 (4.98%)	384 (19.64%)
**Moderate TBI**	**633 (34.16%)**	**549 (29.63%)**	**671 (36.21%)**
BP<90mmHg (initial)	17 (2.69%)	15 (2.73%)	78 (11.62%)
BP<90mmHg (hospital arrival)	10 (1.58%)	7 (1.28%)	47 (7.00%)
**Mild TBI**	**3980 (26.70%)**	**8127 (54.51%)**	**2802 (18.79%)**
BP<90mmHg (initial)	49 (1.23%)	74 (0.91%)	122 (4.35%)
BP<90mmHg (hospital arrival)	19 (0.48%)	34 (0.42%)	83 (2.96%)

**Table 3:** Logistic regression predicting hypotension on hospital arrival (syst BP < 90 mmHg) in patients with TBI, depending on variables like TBI severity, injury pattern, occurrence of initial hypotension, sex and age. Reference categories: no initial hypotension, isolated TBI, mild TBI.

Co-Variable	Odds Ratio	95%-CI	*p*-Value
Initial hypotension yes vs. no (ref = no)	13.82	[11.47; 16.65]	< 0.001
Male vs. Female (ref = female)	0.99	[0.82; 1.21]	0.941
Age (per year increase)	1.01	[1.00; 1.01]	0.001
**Injury pattern**			
Polytrauma vs. isolated (ref = isolated)	3.08	[2.44; 3.90]	< 0.001
Multiple injuries vs. isolated (ref =isolated)	0.73	[0.54; 0.99]	0.045
**TBI severity**			
Severe TBI vs. mild TBI (ref = mild)	4.26	[3.41; 5.32]	< 0.001
Moderate TBI vs. mild TBI (ref = mild)	2.06	[1.49; 2.84]	< 0.001

## Discussion

### Main findings

In this retrospective cohort of adult TBI patients transported by physician-staffed HEMS, hypotension on hospital arrival (SBP < 90 mmHg) was uncommon overall (3.4%) but concentrated in clinically important subgroups. Initial hypotension was the strongest predictor of hypotension on hospital arrival, and severe TBI as well as polytrauma were additional independent predictors. These findings identify a high-risk phenotype: patients who are hypotensive at initial HEMS contact, particularly those with severe TBI and polytrauma, in whom early haemodynamic stabilisation remains a central priority.

### Interpretation and implications

Our analysis does not measure the duration of hypotension and therefore does not support a ‘prolonged’ or ‘persistent’ hypotension construct. Instead, hypotension on hospital arrival should be interpreted as haemodynamic status at handover after prehospital management, which may reflect either ongoing haemodynamic instability, recurrent hypotension during transport, or a failure to achieve sustained stabilisation. In physician-staffed HEMS systems, preventing and correcting hypotension is a central treatment goal; thus, SBP < 90 mmHg on arrival may identify patients in whom stabilisation was not achieved despite advanced prehospital management.

Consistent with emerging international SBP targets, an exploratory SBP < 110 mmHg analysis revealed a similar risk gradient and multivariable pattern of predictors to the primary SBP < 90 mmHg endpoint (Supplementary Tables 1–2), supporting further outcome-linked evaluation of higher prehospital SBP thresholds in high-risk subgroups.

Over recent decades, damage control (DC) strategies for neurotrauma have evolved, particularly in military medicine, yet remain poorly standardised in civilian practice. It is crucial to emphasise that TBI-specific DC strategies differ significantly from those used in general trauma. Notably, permissive hypotension—a concept endorsed in haemorrhagic shock—should be avoided in TBI^13,14^. The Brain Trauma Foundation explicitly advises against permissive hypotension in TBI, recommending higher blood pressure targets (SBP ≥ 100 mmHg)^15^. This approach is supported by DC principles, which advocate early vasopressor use alongside fluid resuscitation^8,16,17^, and is also advocated by the French Guidelines for Severe TBI^18^.

Isotonic crystalloid solutions remain the first-line fluid for prehospital resuscitation. However, the evidence base is limited, and several elements remain under scientific debate^19,20^. Hypotonic fluids, by contrast, should be strictly avoided due to their role in exacerbating vasogenic oedema and their association with increased mortality^21^.

Emerging data suggest that prehospital blood product administration may offer benefits in patients with TBI. Early plasma transfusion has been associated with improved neurological outcomes^22^, and one comparative study showed better results with thawed plasma compared to packed red blood cells as the sole colloid^23^. Some evidence also points to advantages of more liberal transfusion thresholds in this population^24^. While current evidence for whole blood in TBI is not convincing^25^, low-titre group 0 whole blood may provide benefits by delivering a balanced ratio of red cells, plasma, and platelets. Though some studies indicate survival advantages in trauma, the specific impact in TBI requires further targeted research^26^.

### Comparison with existing literature

The detrimental impact of hypotension on outcomes in patients with TBI is well established. Early studies reported up to a 150% increase in mortality among hypotensive TBI patients. Even single episodes of SBP below 90 mmHg in the early phase of care are unequivocally associated with worse neurological outcomes^27^. Early recognition and effective prehospital management of hypotension are therefore critical priorities in TBI care^8^.

Although the optimal resuscitation strategy in trauma remains debated, one principle is clear: patients with TBI—regardless of injury mechanism or associated injuries—require higher minimum SBP targets to preserve cerebral perfusion and prevent secondary brain injury^9,14,28^. Our findings reinforce this by demonstrating that initial hypotension is the strongest predictor of hypotension on hospital arrival, and that patients with severe TBI or polytrauma are especially vulnerable. These high-risk subgroups are unlikely to recover spontaneous haemodynamic stability without targeted intervention. Our data do not evaluate clinical outcomes and therefore cannot be used to recommend specific SBP targets. However, they support the feasibility and importance of identifying high-risk subgroups for future outcome-linked studies of prehospital blood pressure management. Future research should prioritise prospective validation of prehospital protocols focusing on elevated SBP thresholds, early vasopressor use, and evaluation of blood transfusion strategies—particularly in patients with initial hypotension and complex injury patterns.

### Study limitations

This study has important limitations. First, our analysis was restricted to a selected subgroup of adult TBI patients transported by HEMS, so the prevalence estimates are not population based. Second, SBP was available at two discrete time points only; consequently, we cannot infer the duration of hypotension or determine whether hypotension was continuous, intermittent, or newly developed during transport. Third, AIS/ISS, CT findings, bleeding complications, and clinical outcomes (mortality or neurological outcome) were not available in the mission dataset, precluding mechanistic attribution and outcome-based inference. Finally, detailed prehospital time intervals and intervention variables (e.g., fluid volume, vasopressor dosing, blood products) were not sufficiently captured for robust analysis.” This retrospective cohort study relied on existing helicopter emergency medical service (HEMS) mission data, limiting control over variable selection and study design. The absence of a nationwide prehospital registry and strict data protection regulations in Germany, which prohibit linkage with in-hospital outcome data without explicit patient consent, prevented follow-up into clinical endpoints such as mortality or neurological outcome. A total of 4.3% of otherwise eligible patients were excluded due to missing data in one or more key variables. Although this is a relatively low proportion, the possibility of systematic differences between included and excluded patients cannot be entirely ruled out. Key treatment variables such as fluid resuscitation and vasopressor use were incompletely documented, restricting the ability to adjust for their influence on haemodynamic stabilisation. Finally, unmeasured factors such as pre-existing comorbidities, pre-HEMS interventions, or timing of care may have introduced residual confounding.

## Conclusion

Patients presenting with initial hypotension—especially those with severe TBI or polytrauma—are at significantly increased risk of hypotension on hospital arrival. Although our findings may underestimate the true prevalence due to limited measurement points, they nonetheless highlight the need for early and targeted prehospital circulatory management in this high-risk population to mitigate secondary brain injury and improve outcomes.

## Data Availability

The data that support the findings of this study are not publicly available. The data were used upon the approval of the ethical committee and in cooperation with ADAC Luftrettung. However, data are available from the authors upon reasonable request and with permission of ADAC Luftrettung.

## Abbreviations

TBI: traumatic brain injury
SBP: systolic blood pressure
HEMS: helicopter emergency medical service
GCS: Glasgow Coma Scale
OR: Odds Ratio
CI: Confidence Interval
EPIC: Excellence in Prehospital Injury Care project
STROBE: Strengthening the reporting of observational studies in epidemiology
DC: Damage control
